# Vaccination

**Published:** 1882-04

**Authors:** James B. Baird

**Affiliations:** Atlanta, Ga.


ATLANTA

MEDICAL REGISTER.

VOL. I.] APRIL, 1882. [NO. 7

### Original.

### VACCINATION.

BY JAMES B. BAIRD, M.D., ATLANTA, GA.

*Remarks Made Before the Atlanta Medical Society, in Opening the Regular Debate.*

*Mr. President:*

One month ago I was designated as the leader of the debate for this evening, and vaccination was named as the subject which should engage our attention at this meeting. In attempting to discharge the duty imposed by the society I have thought proper to present, very briefly, a few observations, under several subdivisions of the general subject, as best calculated to elicit expressions of opinion upon the part of the gentlemen present, and as furnishing a systematized basis for any discussion that may ensue.

It is unnecessary, before this audience, to offer any arguments in favor of the protective value of vaccination. This fact, of course, is conceded and placed outside the pale of controversy among all men of sufficient intelligence and knowledge to recognize the principle or to appreciate the evidence which underlies and sustains this transcendent discovery of the great and immortal Jenner.

*Virus.*—The illustrious author of vaccination, soon after his doctrine of substituting one disease for the other was promulgated, maintained that cow-pox, once engrafted on the human subject, might be continued from individual to individual by successive transmissions, conferring on each the same immunity from small-pox as was enjoyed by the first one infected direct from the cow, and it is stated upon high authority that the great bulk of vaccinations performed in England at the present day, are done with lymph transmitted from the early direct vaccinations of Jenner himself.

Within a comparatively few years, however, the multiplication of "vaccine farms" in this country has brought the bovine virus into more general use and into prominent notice, and many hasty expressions of opinion, concerning the relative value of humanized and bovine virus, have been indulged. There can be no doubt about the protective qualities of the humanized virus. Experience and observation have established this fact beyond peradventure. But the objection to its use is based upon a fancied deterioration after a series of transmissions through the human subject, and the possible risk of inoculating other diseases with the vaccinia.

The history of the question effectually disposes of the first suggestion, and shows, if the operation is properly performed from a typical and perfectly developed vesical, that its activity and capacity to afford protection from variola is not lost or impaired. The other suggestion will be recurred to in the latter part of these remarks.

Concerning the relative merits of the various forms of humanized virus in common use, it is only necessary to say here that the fresh lymph is generally preferred, but the crust, which is—or ought to be—the inspissated lymph only, yields excellent results and has much to commend it on the score of economy and convenience.

As to the bovine virus, we are enabled to assert, in the first place that no stored virus is as trustworthy as the fresh lymph, and it is practically impossible to employ fresh bovine lymph—that is lymph conveyed directly from the vesicle on the heifer to the arm of the vaccinee.

The bovine virus, as we get it, does undoubtedly very often fail to produce the desired effect, notwithstanding the utmost care and the most conscientious attention to the details of its preparation and insertion—it simply proves inert. In other cases it surely does cause an undesirable and unnecessary amount of local irritation with—and sometimes without—the characteristic results.

These accidents—they may be—can possibly be ascribed to fraud or carelessness in the propagation or collection of the virus, and perhaps a little more honesty, experience and attention may ultimately eliminate these impediments to its successful application. But, at present, speaking for myself, I infinitely prefer to trust my own senses in the choice of a subject and the selection of a vesicle from which to procure lymph, than to accept the *ipse dixit* of any man as to the purity of an article—a commercial commodity—which comes to him through two or three, possibly a dozen, middlemen, and about the quality of which, in the very nature of things, he cannot be positively informed. Then, too, the possibility of transmitting some other infectious disease, along with vaccinia, from the animal to man, must be left for future investigators to determine. This possibility, to say the least, is worthy of thoughtful consideration—more consideration, indeed, than appears to have been bestowed upon it by those persons who so vociferously decry the human product.

*The Operation.*—The precise mode by which the virus is introduced into the system is, perhaps, a question of little moment. Some vaccinators prefer the oblique puncture, thereby depositing the fluid under a little flap of skin. This method is best accomplished by an ordinary thumb lancet, which, to manipulate successfully, must be very sharp. Other operators prefer to scarify, abrade or tattoo the surface, or to make one or more straight incisions or scratches, parallel or crosswise, etc. But the main point is the introduction of the virus in sufficient quantity to produce at least four vesicles, which may be so placed as to result in a discreet or confluent eruption, as the taste of the operator may suggest.

*The Number of Vesicles.*—I have just said the number of vesicles should not be less than four. This statement is based upon evidence furnished by familiar statistics. It is unnecessary to quote those statistics here. Suffice it to say, that the protection afforded by vaccination is in direct ratio with the number of pustules produced up to four, and the utmost care should be observed in primary vaccinations especially, to secure this number at the first trial, as it is a question of grave importance, if the protection is not complete at first, whether the defect can be attoned by revaccinations. It is alleged by competent observers that imperfect primary vaccination does preclude the possibility of ever securing complete protection. In other words, that imperfect primary vaccination may render the system proof against subsequent attempts to produce vaccinia, but it may be, at the same time, insufficient to afford protection against variolous infection. Even if it should be claimed that this view is untenable, it is certainly the part of prudence and wisdom to exercise the most faithful precautions to attain the fullest and best results at the first trial.

*Inspection.*—The popular idea of what constitutes a successful vaccination is very vague, and frequently entirely erroneous. It is commonly thought that the sorest arm evidences the best "take." The arm should invariably be examined by the physician, between the seventh and tenth days, to determine if the evidences of simple, uncomplicated vaccinia are unmistakably present. The neglect of this precaution, upon the part of medical men, is undoubtedly the cause of much of the discredit which has been brought upon vaccination, as many persons who have contracted varioloid have relied for protection upon what they believed to be a successful vaccination, but which, in reality, was no vaccination at all, or so defective as to prove entirely inadequate. This, then, I believe to be a vital point, which cannot be disregarded with safety. The characteristics of the typical vesicle should be clearly pictured in every physician's mind, and any results which do not conform in every particular to the standard, should be discarded and treated as failures.

*Revaccination.* —An important and practical question ofttimes presents itself in reference to the necessity for revaccination. How often shall the operation be repeated? This inquiry may be substantially met by the following reply: A successful vaccination should invariably be secured in infancy or young childhood. The operation should be repeated in youth or early adult life, and in addition to this, revaccination should always be insisted upon wherever there is a liability of exposure to small-pox, or whenever it occurs, or is likely to prevail in any community. In a word, the efficacy of vaccination ought to be verified whenever it appears that it may possibly be subjected to a test. No arbitrary time can, with propriety, be fixed for a repetition of the process, and the above simple rule, it seems to me, is quite sufficient to cover the whole ground.

It is impossible to decide from the appearance presented by the mark or cicatrix how complete is the protection enjoyed by the individual. The only true test is revaccination with absolutely trustworthy virus—and I confess that I am strongly tempted to say eighth day humanized lymph, obtained fresh, directly from the arm of a proper subject.

A certain number of persons appear to possess a remarkable insusceptibility to vaccinia. This circumstance does not insure a corresponding insusceptibility to variola—or, if such insusceptibility should obtain for a time, it may ultimately fail at a critical moment, and, consequently, these subjects should not be abandoned and left to rest confidently in an imaginary security. The apparent insusceptibility may be due to temporary causes—as, for instance, the presence of some unnoticed cutaneous eruption. At all events, the safe and only proper course is the repeated inoculation with unimpeachable vaccine lymph, when, in the vast majority of cases, success, sooner or later, will be achieved.

*The Alleged Dangers of Vaccination.*—It is not incumbent at present to refer to the occasional accidental complications of vaccination. The issue which concerns us is the possibility of transferring other diseases through the medium of the vaccine virus from one subject to another.

So far as I am informed, no medical authority entertains the belief—common among the people—that scrofulous or cutaneous diseases can be transmitted in this way. I shall only advert, therefore, to the so-called vaccinal syphilis, as it is stoutly contended by respectable authority that this disease can be thus propagated.

Some of the most conscientious and careful observers, and some of the most brilliant and distinguished men who ever graced the medical profession deny *in toto* the possibility of invaccinating any disease whatever, and they have grouped together and analyzed an enormous amount of material—thousands upon thousands of cases—to prove that such invaccination of disease is impossible.

Dr. Edward Cator Seaton says, in Reynold's System of Medicine, "Direct experiments made on a large scale, at many times, and by many individuals, have led in every single instance to the same conclusion. M. Cullerier, and other experimenters in France, especially M. Taupin, have taken lymph on purpose from syphilitic children, have vaccinated healthy children with it, and watched the result. In no instance has syphilis been communicated. Heim made similar experiments in Germany with the same result. It was found no more possible to produce syphilis by vaccine lymph taken from a syphilitic child, than it is to produce small-pox by lymph taken from vaccine vesicles on the arms of patients who are incubating, or suffering from that disease. This, it is well known, has been done hundreds of times, but never has small-pox been thus communicated, or anything but a vaccine vesicle resulted." Mr. Marson "has never seen other diseases communicated with the vaccine disease, nor does he believe in the popular report that they are so communicated." This was the experience also of Mr. Leese, Sir W Jenner, Dr. West and Sir James Paget.

Dr. West declaring "that there has never come under his notice any instance in which there seemed the slightest pretext for supposing that syphilis had been communicated to infants through the medium of vaccine lymph."

But I refrain from multiplying evidence of this character. If these witnesses are discredited the skeptic would not be convinced though one rose from the dead. Nevertheless, let me emphasize one point, namely, that the extreme improbability of inoculating other diseases with the vaccinia will not excuse carelessness or looseness in the selection of lymph, and, while I would condemn recklessness and foolhardy indifference to what seems to be a groundless apprehension, on the one hand, I would just as strenuously oppose a cowardly acquiescence, upon the part of the physician, in an unfounded popular prejudice against humanized virus on the other.

This proposition at least I feel safe in asserting: In view of the fact that many millions of human creatures have been vaccinated during the last eighty-five years in all parts of the world, if the danger of transferring other diseases with the cow-pox, from one individual to another, had been more than infinitesimal, it would long ago have been established, and ceased, ere this, to be a question open to discussion.

